# IL-6 and IL-17 as potential links between pre-existing hypertension and long-term COVID sequelae in patients undergoing hemodialysis: a multicenter cross-sectional study

**DOI:** 10.1038/s41598-024-54930-z

**Published:** 2024-02-29

**Authors:** Natalia Stepanova, Victoria Driianska, Andriy Rysyev, Tetyana Ostapenko, Nataliia Kalinina

**Affiliations:** 1 Department of Nephrology and Dialysis, State Institution “Institute of Nephrology of the National Academy of Medical Sciences”, Kyiv, Ukraine; 2 Laboratory of Immunology, State Institution “Institute of Nephrology of the National Academy of Medical Sciences”, Kyiv, Ukraine; 3 Dialysis Medical Center LLC “Link-Medital”, Odesa, Ukraine; 4 Dialysis Medical Center LLC “Nephrocenter”, Zaporizhzhia, Ukraine

**Keywords:** Renal replacement therapy, Hypertension, Medical research, Nephrology

## Abstract

Long COVID, characterized by persistent symptoms following acute infection, poses a significant health challenge, particularly for patients with pre-existing chronic conditions such as hypertension. We hypothesized that an increase in the production of interleukins (IL)-6 and IL-17 could serve as a potential mechanism linking pre-existing uncontrolled blood pressure (BP) to the occurrence of long-term COVID sequelae in patients undergoing hemodialysis (HD). This cross-sectional study examined serum IL-6 and IL-17 levels in 80 patients undergoing HD, considering preinfection BP, the presence of long-term COVID sequelae, and the time interval after acute COVID-19 infection, which was either 5 or 10 months. Controlled BP was defined as a 3-month average pre-dialysis BP < 140/90 mmHg and post-dialysis < 130/80 mmHg. The findings suggest that the prevalence of long-term COVID sequelae was significantly higher in patients with uncontrolled BP than in the BP-controlled group. Both IL-6 and IL-17 concentrations were also significantly higher in patients with uncontrolled BP compared with the BP-controlled group. The patients with long-term COVID sequelae had higher IL-6 and IL-17 values than the fully recovered patients at both time points, but their concentrations decreased significantly over time. Further research and prospective studies are warranted to validate these findings.

## Introduction

Patients undergoing maintenance hemodialysis (HD) are at high risk of becoming infected and experiencing severe complications from coronavirus disease 2019 (COVID-19)^1,2^. Older age, immunosuppressed status, and comorbidity burden increase COVID-19-associated mortality 20-fold in patients undergoing HD compared with the general population^1,3^. However, the problem of COVID-19 in patients treated with HD is not limited to the acute phase of the disease, and the persistence of long-term COVID sequelae has been reported in 81–98% of them^4,5^.

Hypertension, a nearly ubiquitous complication in patients undergoing HD, has been identified as a risk factor for COVID-19 and is hypothesized to be a potential risk factor for long COVID^6^. IL-6 and IL-17 have been shown to play an important role in both the severity of COVID-19^7,8^ and the promotion of hypertension^9,10^. However, the evidence supporting the role of IL-6 and IL-17 in the development of long COVID within the general population is limited^11,12^, and only one study has focused on analyzing cytokine levels specifically in individuals undergoing HD who experience long-term COVID sequelae^13^. We hypothesized that an increase in the production of IL-6 and IL-17 could serve as a potential mechanism linking pre-existing uncontrolled blood pressure (BP) to the occurrence of long-term COVID sequelae in patients undergoing HD. Consequently, this study sought to investigate serum levels of IL-6 and IL-17, considering variables such as preinfection BP, the presence of long-term COVID sequelae, and the duration following acute COVID-19 infection.

## Results

The study sample consisted of 80 patients undergoing HD aged 56 (44–63.2) years with a dialysis vintage of 4.7 (3.3–7.08) years. Among these patients, 45 (56.2%) were males, 11 (13.75%) were obese, 19 (23.7%) had anemia, and 22 (27.5%) had mineral and bone disorders. All patients exhibited arterial hypertension, but 46 (57.5%) had uncontrolled BP before infection with COVID-19. The predominant contributors to inadequate BP management were delineated as volume overload in 19 (41.3%) patients and suboptimal adherence to antihypertensive medications in 11 (23.9%) patients due to noncompliance. Additionally, 3 patients (6.5%) opted to withhold their medications preceding dialysis due to a history of intradialytic hypotension, while 13 individuals (28.2%) encountered resistant hypertension despite being prescribed three or more antihypertensive agents from diverse therapeutic classes.

Patients with uncontrolled BP were more likely to have hypertension as the primary cause of their end-stage kidney disease (patients with diabetes were excluded from the study), had lower Kt/V values, and tended to be overweight compared to those with controlled BP. However, no significant differences were observed between the two groups in terms of other routine laboratory tests and medications taken (Table 1). Moreover, among patients with preexisting uncontrolled BP, a higher prevalence of hospitalization with oxygen support was noted during the acute phase of COVID-19. Nevertheless, no significant differences were found in the prevalence of asymptomatic and mild to moderate severity compared to the controlled BP group. Notably, a significant difference was observed in the prevalence of long COVID between the uncontrolled BP group and the group with controlled BP (see Table 1).

**Table 1:** Characteristics of the patients included in the study.

	Uncontrolled BP (n = 46)	Controlled BP (n = 34)	*p-*value
Demographic data			
Male gender, n (%)	28 (60.9%)	17 (50%)	0.34
Age, years	56 (44–62)	55 (45–64)	0.90
Causes of ESKD			
Hypertension, n (%)	27 (58.7%)	12 (35.3%)	0.04
Primary glomerulonephritis	9 (19.6%)	11 (32.4%)	0.19
Polycystic kidney disease	4 (8.7%)	3 (8. %)	0.98
Interstitial nephritis	2 (4.3%)	2 (5.9%)	0.75
Other	4 (8.7%)	6 (17,6%)	0.24
Routine clinical data			
Dialysis vintage, years	5.2 (3.4–10.2)	4.3 (3.3–5.2)	0.11
Long COVID, n (%)	31 (67.4%)	11 (32.4%)	0.002
BMI, kg/m^2^	27.9 (24.2–31.3)	25.6 (23.3–29.8)	0.07
Kt/V	1.3 (1.3–1.4)	1.4 (1.3–1.5)	0.003
IDWG, kg	2.4 (1.8–3.0)	2.0 (1.5–3.0)	0.52
Systolic blood pressure, mm Hg	145 (140–160)	120 (117.5–130)	< 0.0001
Diastolic blood pressure, mm Hg	85 (80–95)	70 (60–80)	< 0.0001
Hb, g/L	98.4 (91.2–117)	107 (94.2–113)	0.57
Serum albumin, g/L	38.5 (36.1–41.6)	36.9 (35.5–41.7)	0.62
Calcium, mmol/L	2.32 (2.22–2.36)	2.31 (2.24–2.40)	0.62
Phosphorus, mmol/L	1.69 (1.35–1.92)	1.51 (1.25–2.15)	0.59
iPTH, ng/L	150 (110–389)	168 (77.4–656.7)	0.59
Total cholesterol, mmol/L	4.9 (4.2–5.6)	4.7 (4.4–5.2)	0.76
CRP, mg/L	10.4 (8.6–15.1)	9.7 (6.9–14.8)	0.87
Ferritin (ng/ml)	303 (159.3–376.5)	322 (135.5–401)	0.21
Medications			
ACE inhibitors/RAAS blockers, n (%)	32 (69.6%)	19 (55.9%)	0.21
Beta blockers, n (%)	37 (80.4%)	22 (64.7%)	0.12
Calcium channel blockers, n (%)	32 (69.6%)	17 (50.0%)	0.07
Alpha blockers, n (%)	11 (23.9%)	3 (8.8%)	0.08
Diuretics, n (%)	6 (13.0%)	2 (5.9%)	0.29
Iron supplementation, n (%)	19 (41.3%)	18 (52.9%)	0.31
Erythropoietins, n (%)	40 (86.9%)	28 (82.3%)	0.57
Non-calcium phosphate binders, n (%)	18 (39.1%)	10 (29.4%)	0.37
Acute COVID-19 severity			
Asymptomatic COVID-19, n (%)	6 (13.0%)	9 (26.5%)	0.12
Mild to moderate COVID-19, n (%)	25 (54.3%)	21 (61.8%)	0.50
Hospitalization with oxygen supply, n (%)	15 (32.7%)	4 (11.8%)	0.03

Abbreviation: ACE—angiotensin-converting enzyme, BMI—body mass index, CRP—C-reactive protein, Hb—hemoglobin, IDWG—interdialytic weight gain, iPTH—intact parathyroid hormone, RAAS—renin–angiotensin–aldosterone system.

**Figure 1:**
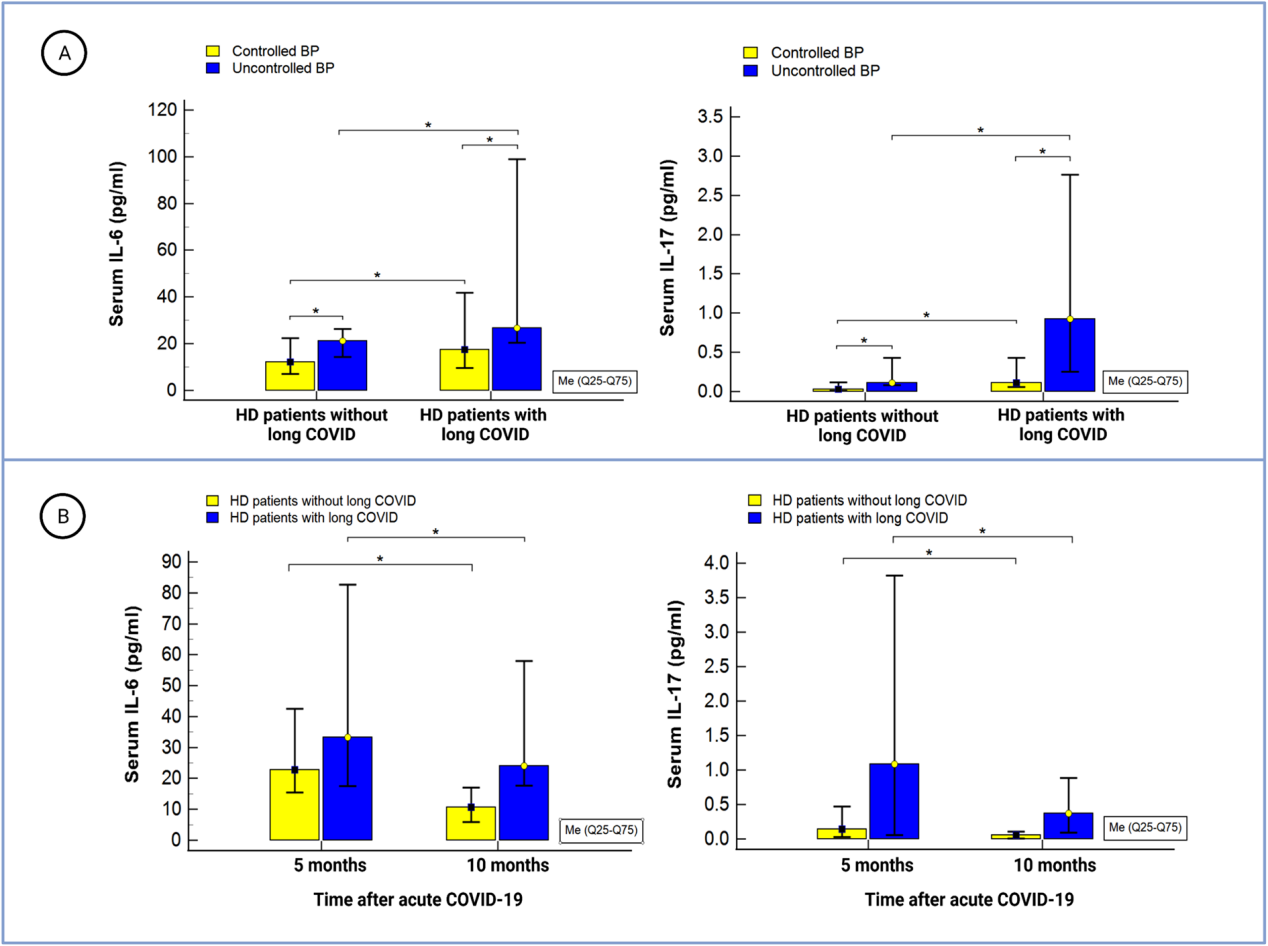
(**A**): IL-6 and IL-17 serum concentrations in HD patients stratified by preinfection BP and the presence of long-term COVID sequelae. (**B**): IL-6 and IL-17 serum concentrations in HD patients stratified by the presence of long-COVID sequelae and the interval between acute COVID-19 and blood collection. Significance was determined by mixed-effects ANOVA and Tukey’s post hoc analysis; (*): *p* < 0.001. Abbreviations: BP—blood pressure; HD—hemodialysis; IL—interleukin.

Both IL-6 and IL-17 serum concentrations were significantly higher in patients undergoing HD compared to the control group: 21.3 (12.8–40.7) vs 5.8 (1.9–10.7) pg/mL, *p* = 0.0001 and 0.11 (0.03–0.68) *vs*. 0.055 (0.01–0.66) pg/mL, *p* = 0.02, respectively. Upon stratification by pre-existing BP status, significantly higher cytokine concentrations were observed in the BP-uncontrolled group compared to the BP-controlled group (Fig. 1A).

Notably, patients with both pre-existing uncontrolled BP and long-term COVID sequelae demonstrated the highest levels of IL-6 and IL-17. Additionally, patients with long-term COVID sequelae exhibited significantly higher cytokine levels compared to fully recovered patients at both time points following acute COVID-19 infection (Fig. 1B). Remarkably, the concentrations of IL-6 and IL-17 exhibited a pronounced decrease over time. Comprehensive statistical details on the differences observed among the studied subgroups are provided in the Supplementary file, Table S1.

The Spearman correlation test, encompassing all demographic and laboratory markers within the entire patient cohort, revealed a positive correlation between IL-6 and interdialytic weight gain (IDWG) (r = 0.35, *p* = 0.005), and showed a positive correlation with serum phosphate levels (r = 0.28, *p* = 0.025) (Supplementary file, Table S2). Notably, in the overall study cohort, IL-6 and IL-17 concentrations were not linked to systolic or diastolic blood pressure (see Table S2). However, upon analyzing the subgroup of patients with long COVID separately, IL-6 exhibited a direct association with diastolic blood pressure (r = 0.39, *p* = 0.019), while IL-17 showed an association with systolic blood pressure (r = 0.47, *p* = 0.004).

To control for potential confounding factors affecting cytokine levels, a partial correlation analysis was conducted, incorporating patients' age, dialysis vintage, and all statistically significant variables as covariates. The results of the partial correlation test indicated that both serum IL-6 (r = 0.29, *p* = 0.048) and IL-17 (r = 0.33, *p* = 0.003) concentrations were significantly associated with long-term COVID sequelae, independently of patients' age, sex, dialysis vintage, BMI, Kt/V, IDWG, serum phosphate levels, and acute COVID-19 severity.

To validate the significant differences in cytokine concentrations obtained in the studied subgroups, a sensitivity analysis was performed using two-way ANOVA with Tukey’s test after applying the Box-Cox transformation to the cytokine data. The analysis, which considered pre-existing BP status, the presence of long-term COVID sequelae, and time points following COVID-19 as additional factors, showed that the differences observed for IL-6 and IL-17 remained significant. Specifically, there was a significant main effect of pre-existing uncontrolled BP on IL-6 (F = 5.9, *p* = 0.018) and IL-17 (F = 4.6, *p* = 0.036), as well as a significant main effect of long-term COVID sequelae on IL-6 (F = 5.6, *p* = 0.002) and IL-17 (F = 8.6, *p* = 0.004) concentrations. Furthermore, the effect of the time interval after acute COVID-19 was also significant for both IL-6 (F = 9.4, *p* = 0.003) and IL-17 concentrations (F = 6.12, *p* = 0.016) (Supplementary file, Table S3).

## Discussion

The impact of pre-existing hypertension on the development of severe COVID-19 outcomes has been extensively studied in the general population^14,15^. In HD patients, despite the 58% failure rate in reaching target BP levels that increase vulnerability to COVID-19 complications, the association between pre-existing BP and long COVID remains a research gap^16^. To address this issue, we explored proposed mechanisms linking long COVID and the cardiovascular system, such as chronic inflammation with elevated levels of chemokines, cytokines, angiotensin II, and endothelial dysfunction^17^. We hypothesized that these mechanisms may also apply to HD patients and focused our study on analyzing serum IL-6 and IL-17 concentrations as potential links between uncontrolled BP and long COVID. The study revealed that both uncontrolled hypertension and long COVID resulted in elevated IL-6 and IL-17 concentrations, peaking in patients with both conditions and decreasing over time after infection, possibly reflecting Th17-related systemic inflammation during the acute phase of COVID-19. Consistent with our findings, elevated IL-6^11,12,18^ and IL-17^19^ levels have been reported in a general cohort of patients with long-term COVID sequelae, and the only study by Corrêa et al. studied cytokine levels as long COVID predictors in patients undergoing HD^13^. However, the authors did not assess pre-infection BP status and found no IL-6 increase in their cohort.

Current studies focus on cytokine profiling in patients with long COVID, showing persistent deregulation of cytokines post-infection^11,19–21^. However, the unique characteristics of patients undergoing HD, including a variety of comorbidities, uremic environment, impaired immune responses, chronic inflammation, and HD dose and vintage, significantly alter the immune response to COVID-19^22–25^. In contrast to the general population of COVID-infected patients, the baseline chronic inflammation in patients undergoing HD prevents a sustained increase in cytokines^23,26^. This discrepancy suggests that the accumulation of cytokines and chemokines during COVID-19 is primarily attributed to impaired renal clearance in HD patients^27^. Our findings support the impact of these unique characteristics on long-term immune-related COVID-19 outcomes in HD patients, shown by significant differences in IL-6 and IL-17 concentrations between convalescent HD patients and non-CKD controls. Uremic toxins can directly impact immune cell function, creating an inflammatory environment and upregulating pro-inflammatory cytokine production following a COVID-19 infection^28,29^.

As mentioned above, several shared mechanisms, including chronic inflammation, activation of the renin–angiotensin–aldosterone system (RAAS), and endothelial dysfunction, might explain the association between pre-existing uncontrolled BP, high IL-6, and IL-17 concentrations, and long COVID in patients undergoing HD. Uremia-related inflammation with increased serum cytokine levels is a well-known phenomenon in patients undergoing HD^22,30,31^. Hypertension may also raise IL-6 and IL-17 levels, contributing to chronic inflammation^32,33^. Therefore, it is logical to assume that elevated IL-6 and IL-17 production in hypertensive patients undergoing HD may be further stimulated by persistent SARS-CoV-2 or by cytokines/chemokines produced by other immune cells, resulting in a dysregulated immune response and long COVID.

Chronic activation of the RAAS might be another potential route linking hypertension to long COVID development. Hypertension causes RAAS dysregulation, resulting in higher angiotensin II levels, which then promote inflammation, oxidative stress, and vascular dysfunction^34^. These processes can contribute to the persistence of COVID-19 symptoms and the development of long COVID in hypertensive individuals^35^. In turn, SARS-CoV-2 binds to ACE2 receptors and may disrupt the RAAS system^36^, potentially causing increased cytokine production and a hyperinflammatory response in HD patients with long COVID. In line with this hypothesis, elevated plasma ACE2 activity was observed months after COVID-19 compared to similar uninfected controls^37^. Moreover, in animal models, angiotensin II has been shown to stimulate the production of IL-6 and IL-17^38,39^, and this mechanism may be relevant in humans^40,41^.

Furthermore, both hypertension and COVID-19 are associated with endothelial dysfunction^42,43^, which may increase adhesion molecules and proinflammatory cytokines, including IL-6 and IL-17. SARS-CoV-2 infection disrupts endothelial balance, raising oxidative stress and reducing NO bioavailability^43^. This acute oxidative damage predicts long-term COVID sequelae 3–4 months later^44^. Although COVID-19 patients may experience a gradual improvement in endothelial dysfunction over a 6-month follow-up, their endothelial function remains impaired compared to healthy individuals^45^.

Our study possesses several notable limitations that necessitate acknowledgment. The main limitations of our study are its cross-sectional design and the relatively small sample size; therefore, our findings only revealed associations and causality could not be established. In addition, even though we meticulously selected patients and ensured that those with controlled and uncontrolled BP had similar characteristics, we cannot entirely dismiss the potential impact of various confounding immune-related factors within each subgroup under analysis. Variables like obesity, mineral and bone disorders, or anemia, which have the potential to induce chronic inflammation, could contribute to elevated cytokine levels. Finally, it is important to acknowledge the limitations associated with the cytokine measurement method, including potential interference or cross-reactivity of the assays, the influence of biological variability, and potential variability introduced during sample transportation to the laboratory.

Notwithstanding these limitations, our study is the first to report a potential association between elevated IL-6 and IL-17 serum concentrations with pre-existing uncontrolled BP and the development of long-term COVID sequelae in patients undergoing HD. Further research is warranted to fully understand the mechanisms underlying this association and to develop effective treatments for long COVID.

## Methods

### Study design

This multicenter cross-sectional study was part of the ongoing project "Mechanisms of Development and Therapeutic Targets of Post-COVID Syndrome in Dialysis Patients" (National Study Registration Number 0122U000144) of the State Institution “Institute of Nephrology of the National Academy of Medical Science of Ukraine”, Kyiv, Ukraine. The study was conducted in accordance with the Declaration of Helsinki and was carried out between November 2021 and May 2022. The Institute Ethics Committee approved the study protocol (protocol number: 2–2021, dated April 6, 2021). Written informed consent was obtained from all patients before enrollment.

### Sample size

The required sample size was estimated using G*Power software (version 3.1.9.7) based on a previously published study of cytokine measurement in patients undergoing HD following COVID-19 infection^13^. The authors reported effect sizes of 0.81 based on sample sizes of 64 for the long-COVID group and 13 for the non-long-COVID group. Considering this effect size (Cohen's d = 0.81), an alpha level of 0.05, and a power of 0.80, we determined that a minimum sample size of 34 participants in each group would be required to detect statistical differences using both Mann–Whitney test and the ANOVA main effects and interactions test.

### Study participants

**Figure 2:**
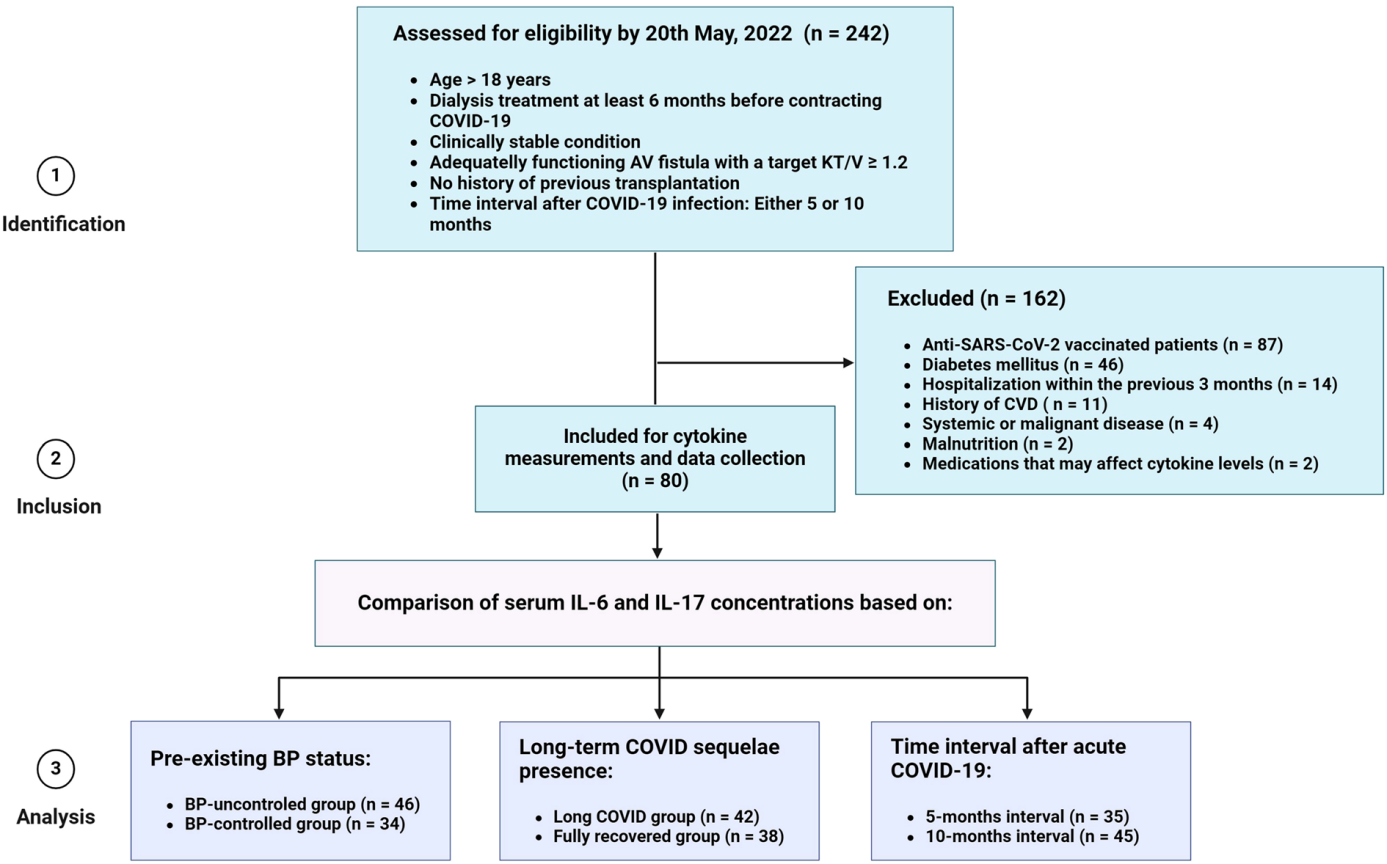
Flow diagram of the study design. The flowchart provides an overview of the selection process to ensure that the final cohort of patients is an appropriate population to study the research question. CVD included angina, myocardial infarction, stroke, heart failure, or peripheral artery disease requiring hospitalization (abbreviated as a history of CVD in the figure). Medications that may affect cytokine levels include glucocorticoids, tocilizumab, nonsteroidal anti-inflammatory drugs, or thalidomide. Abbreviations: AV—arteriovenous, BP—blood pressure, CVD—cardiovascular disease, IL—interleukin.

A total of 80 HD patients who had experienced COVID-19 at least 5 months before enrollment were included in this study. The patients were recruited from three dialysis centers located in different regions of Ukraine (Kyiv, Odesa, and Zaporizhzhia) based on inclusion/exclusion criteria and the time interval after COVID-19 infection, which was either 5 or 10 months (Fig. 2).

This patient selection approach enabled the examination of cytokine levels at two distinct time points, the analysis of potential changes over time, and minimized the potential impact of time-related variations in cytokine levels.

All the patients were dialyzed three times a week for 4 h using the Fresenius 5008S High Volume HDF System. Patient clinical records were examined upon study entry to gather pre-infection BP and supplementary information. Controlled BP was characterized by an average pre-dialysis BP of less than 140/90 mmHg and a post-dialysis BP of less than 130/80 mmHg over a three-month period. The severity of acute COVID-19 was categorized into three groups: asymptomatic (no observable symptoms of COVID-19 with a positive PCR test), mild to moderate (manifestation of COVID-19 symptoms or pneumonia without the need for oxygen support), and requiring hospitalization with oxygen support. Long COVID was diagnosed based on the presence of at least one clinical symptom in patients following their COVID-19 infection, which could not be attributed to any other known medical condition or disease^46^.

### Cytokine measurements

IL-6 and IL-17 testing was conducted using the "SunRise TouchScreen" enzyme and commercially available ELISA kits (IBL International GmbH, Hamburg, Germany). The patients fasted overnight and avoided strenuous activity for 24 h before blood collection. Predialysis blood samples (5 mL) were collected, centrifuged at 1500 rpm for 10 min to separate plasma/serum, and stored at −20 °C until assay. Cytokine analysis followed the manufacturer’s protocol, with samples run in duplicate. The minimum detectable dose for the quantification of IL-6 was 0.6 pg/mL, and the standards ranged from 0.3 to 2560 pg/mL. As for IL-17, the minimum detectable dose was 0.01 ng/mL, and the standards ranged from 0.01 to 100 pg/mL. The ELISA reader used for the measurements was the Tecan SunriseTM Absorbance Microplate Reader. The overall inter-assay coefficient of variation was 7.1% for IL-6 and 9.1% for IL-17.

To highlight the inflammatory state of patients undergoing HD and establish the cytokines reference levels in the general cohort of COVID-19 convalescents at the same time frame, serum samples were also collected from 20 volunteers. The volunteers were matched with the study group in sex (55% male) and age (51 (43–56) vs. 56 (44–63) years, *p* = 0.08). They did not have chronic kidney disease or uncontrolled BP but had previously contracted COVID-19 at the same time point and did not exhibit long-term COVID sequelae.

### Statistical analysis

The statistical analysis and graphs were performed using MedCalc Statistical Software version 20.218 (MedCalc Software Ltd., Ostend, Belgium). Demographic and clinical data were expressed as proportions or medians (Me) and interquartile ranges (Q25–Q75) and compared with the Chi-squared test (χ^2^) or the Mann–Whitney U test, as appropriate. The Kruskal–Wallis test with the Dunn post-hoc test was employed to assess the interaction effects of pre-existing uncontrolled BP, long-term COVID sequelae, and time after acute COVID-19 on cytokine levels. The association between cytokine levels and clinical and laboratory markers was analyzed using the Spearman correlation test, and partial correlation was utilized to adjust for the confounding impact of studied markers on the association serum cytokine concentrations. Finally, a two-way ANOVA with Tukey’s multiple comparisons test was used as a sensitivity analysis due to the small sample sizes in some subgroups. To normalize the distribution of cytokine data and mitigate any biases associated with extreme values, a Box–Cox transformation function was applied.

### Ethical approval and consent to participate

The study was conducted in accordance with the Declaration of Helsinki and was carried out between November 2021 and May 2022. The study protocol was approved by the Ethics Committee of the State Institution "Institute of Nephrology of the National Academy of Medical Sciences", Kyiv, Ukraine (protocol number: 2–2021, dated April 6, 2021). Written, informed consent was obtained from all patients before enrollment.

### Supplementary Information

Supplementary Information.

## Data Availability

The data used in the study are available upon reasonable request to the corresponding author.
