# Supplementary material for: IL-6 and IL-17 as potential links between pre-existing hypertension and long-term COVID sequelae in patients undergoing hemodialysis: a multicenter cross-sectional study
Source: Sci Rep. 2024 Feb 29;14:4968. doi: 10.1038/s41598-024-54930-z (PMC10904824; doi:10.1038/s41598-024-54930-z)
Supplement: Supplementary file 1 — Supplementary Information. [file 41598_2024_54930_MOESM1_ESM.pdf]

Natalia Stepanova, Victoria Driianska, Andriy Rysyev, Tetyana Ostapenko, Nataliia Kalinina

IL-6 and IL-17 as potential links between pre-existing hypertension and long-COVID sequelae in patients undergoing hemodialysis: A multicenter cross-sectional study

Supplementary file

Table S1. Kruskal-Wallis Test with Dunn Post-hoc Analysis

Cytokine, pg/mL	Variable	Obs. without missing data	25th percentile	Median	75th percentile	p-value
Controlled BP (n = 34)						
IL-6	Patients with long-term COVID sequelae	11	9.95	18.60	41.40	0.003
	Patients without long-term COVID sequelae	23	7.05	11.60	21.98	
IL-17	Patients with long-term COVID sequelae	11	0.045	0.14	0.40	0.024
	Patients without long-term COVID sequelae	23	0.013	0.030	0.095	
Uncontrolled BP (n = 46)						
IL-6	Patients with long-term COVID sequelae	31	17.72	29.40	73.13	0.049
	Patients without long-term COVID sequelae	15	12.10	20.35	27.10	
IL-17	Patients with long-term COVID sequelae	31	0.17	0.88	2.66	0.001
	Patients without long-term COVID sequelae	15	0.020	0.11	0.30	
Patients without long-term COVID sequelae (n = 38)						
IL-6	5-month interval after COVID-19	13	12.1	18.4	27.1	0.0001
	10-month interval after COVID-19	25	2.90	3.90	5.80	
IL-17	5-month interval after COVID-19	13	0.02	0.03	0.16	0.04
	10-month interval after COVID-19	25	0.01	0.015	0.06	
Patients with long-term COVID sequelae (n = 42)						
IL-6	5-month interval after COVID-19	32	21.5	39.1	90.3	0.03
	10-month interval after COVID-19	10	13.1	22.3	47.3	
IL-17	5-month interval after COVID-19	32	0.68	1.2	1.3	0.001
	10-month interval after COVID-19	10	0.06	0.11	0.44	

Table S2. Correlation matrix (Spearman)

Variables	IL-6, pg/mL	IL-17, pg/mL	Kt/V	Hb, g/L	Age, years	BMI, kg/m^2	Dialysis duration, years	IDWG, kg	CRP, mg/L	Calcium, mmol/L	Phosphorus, mmol/L	Systolic blood pressure, mm Hg	Diastolic blood pressure, mm Hg
**IL-6, pg/mL**	**1**	**0.354**	-0.095	-0.018	0.093	0.118	-0.147	**0.348**	0.078	-0.053	-0.091	0.143	0.185
**IL-17, pg/mL**	**0.354**	**1**	0.198	0.075	0.163	0.014	0.121	0.233	0.058	0.045	**0.282**	0.188	0.031
**Kt/V**	-0.095	0.198	**1**	0.234	0.180	**-0.372**	-0.009	-0.023	**-0.255**	**0.430**	0.044	-0.054	-0.248
**Hb, g/L**	-0.018	0.075	0.234	**1**	-0.139	0.021	0.054	0.078	**-0.371**	0.070	0.209	-0.242	-0.221
**Age, years**	0.093	0.163	0.180	-0.139	**1**	0.171	**0.343**	**-0.379**	0.077	0.172	**-0.465**	**0.320**	0.008
**BMI, kg/m^2**	0.118	0.014	**-0.372**	0.021	0.171	**1**	0.013	**0.305**	-0.109	-0.115	-0.084	-0.130	0.232
**Dialysis duration, years**	-0.147	0.121	-0.009	0.054	**0.343**	0.013	**1**	-0.108	0.187	0.152	-0.154	0.043	**-0.294**
**IDWG, kg**	**0.348**	0.233	-0.023	0.078	**-0.379**	**0.305**	-0.108	**1**	-0.066	-0.184	**0.408**	**0.250**	0.086
**CRP, mg/L**	0.078	0.058	**-0.255**	**-0.371**	0.077	-0.109	0.187	0.066	**1**	0.195	**0.437**	0.111	0.009
**Calcium, mmol/L**	-0.053	0.045	**0.430**	0.070	0.172	-0.115	0.152	-0.184	0.195	**1**	**-0.316**	0.043	**-0.470**
**Phosphorus, mmol/L**	-0.091	**0.282**	0.044	0.209	**-0.465**	-0.084	-0.154	**0.408**	**0.437**	**-0.316**	**1**	-0.060	0.074
**Systolic blood pressure, mm Hg**	0.143	0.188	-0.054	-0.242	**0.320**	-0.130	0.043	**0.250**	0.111	0.043	-0.060	**1**	**0.286**
**Diastolic blood pressure, mm Hg**	0.185	0.031	-0.248	-0.221	0.008	0.232	**-0.294**	0.086	0.009	**-0.470**	0.074	**0.286**	**1**
Values in bold are different from 0 with a significance level of alpha=0.05													

Abbreviation: BMI – body mass index, CRP – C-reactive protein, Hb – hemoglobin, IDWG - interdialytic weight gain, iPTH – intact parathyroid hormone.

Table S3. Two-Way ANOVA with Post-hoc Analysis Using Tukey's Multiple Comparisons Test

Summary statistics (Quantitative data):

Variable	Observations	Obs. without missing data	Minimum	Maximum	Mean	Std. deviation
IL-6t	80	80	0.637	4.810	2.919	0.920
IL-17t	80	80	0.010	2.380	0.054	0.023

t – Box-Cox transformed data

Summary statistics (Qualitative data):

Variable	Categories	Counts	Frequencies	%
Pre-existing uncontrolled BP	0	34	31	42.5
	1	46	46	57.5
Long COVID	0	38	37	47.5
	1	42	40	52.5
Time Interval	5 m	33	30	41.2
	10 m	47	47	58.7

Summary of the normality tests for all the dependent variables:

	p-value
IL_6t	0.581
IL_17t	0.250

The sum of Squares Analysis (IL-6t):

Source	DF	Sum of squares	Mean squares	F	Pr > F	p-values signification codes
Pre-existing uncontrolled BP	1.000	3.775	3.775	5.905	**0.018**	*
Long COVID	1.000	3.602	3.602	5.634	**0.020**	*
Time Interval	1.000	6.014	6.014	9.407	**0.003**	**

*Signification codes: 0 < *** < 0.001 < ** < 0.01 < * < 0.05 < . < 0.1 < ° < 1*

The sum of Squares Analysis (IL-17t):

Source	DF	Sum of squares	Mean squares	F	Pr > F	p-values signification codes
Pre-existing uncontrolled BP	1.000	18.312	18.312	4.557	**0.036**	*
Long COVID	1.000	34.702	34.702	8.635	**0.004**	**
Time Interval	1.000	24.613	24.613	6.125	**0.016**	*

*Signification codes: 0 < *** < 0.001 < ** < 0.01 < * < 0.05 < . < 0.1 < ° < 1*
